# Viral hepatitis-associated intrahepatic cholangiocarcinoma shares common disease processes with hepatocellular carcinoma

**DOI:** 10.1038/sj.bjc.6605063

**Published:** 2009-05-12

**Authors:** C H Lee, C J Chang, Y J Lin, C N Yeh, M F Chen, S Y Hsieh

**Affiliations:** 1 Division of General and Geriatric Medicine, Chang Gung Memorial Hospital, Taoyuan, Taiwan; 2 Resource Center for Clinical Research, Chang Gung Memorial Hospital, Taoyuan, Taiwan; 3 Department of General Surgery, Chang Gung Memorial Hospital, Taoyuan, Taiwan; 4 Chang Gung University School of Medicine, Taoyuan, Taiwan; 5 Liver Research Unit, Chang Gung Memorial Hospital, Taoyuan, Taiwan

**Keywords:** cholangiocarcinoma, hepatocellular carcinoma, viral hepatitis, hepatic progenitor cells, combined hepatocellular cholangiocarcinoma

## Abstract

Bile duct cells and hepatocytes differentiate from the same hepatic progenitor cells. To investigate the possible association of viral hepatitis B and C with intrahepatic cholangiocarcinoma (ICC), we conducted a retrospective case–control study using univariate and multivariate logistic analyses to identify risk factors for ICC. Besides hepatic lithiasis (25.6%; *P*<0.001), seropositivity for hepatitis B surface antigen (37.5% of all ICC patients; odds ratio (OR) =4.985, *P*<0.001) and seropositivity for hepatitis C antibodies (13.1%; OR=2.709; *P*=0.021) are the primary independent risk factors for ICC. Cirrhosis exerted synergic effects on the development of ICC. We compared the age distributions of viral-hepatitis associated ICC to that of viral hepatitis-associated hepatocellular carcinoma (HCC). The mean age of ICC patients with viral hepatitis B (56.4±11.1 years) were 9 years younger than that of ICC patients with viral hepatitis C (65.6±9.17 years), similar to that observed in HCC. The incidence ratio of HCC : ICC : CHC (combined hepatocellular cholangiocarcinoma) in our population was 233 : 17 : 1 consistent with the theoretic ratio of hepatocyte number to cholangiocyte number in the liver. Our findings indicated that both viral hepatitis-associated ICC and HCC shared common disease process for carcinogenesis and, possibly, both arose from the hepatic progenitor cells.

Primary liver cancer is one of the most common cancers in the world. Intrahepatic cholangiocarcinoma (ICC) is the second most frequent primary liver cancer after hepatocellular carcinoma (HCC), which accounts for more than 80% of these cases. Combined hepatocellular cholangiocarcinoma (CHC) is also occasionally found, but is relatively rare with a frequency of ∼1% of the total primary liver cancers (Bhagat *et al*, 2006). Hepatocellular carcinoma is strongly associated with chronic viral hepatitis B and C and liver cirrhosis. In addition, other forms of chronic inflammation of the liver including alcoholic liver disease, hemochromomatosis and congenital and acquired metabolic diseases also contribute to the development of HCC (El-Serag and Rudolph, 2007). On the other hand, the etiology of ICC remains unclear (Shaib *et al*, 2007; Welzel *et al*, 2007; Blechacz and Gores, 2008b). Though several risk factors, including hepatolithiasis, inflammatory bile duct disease (sclerosing cholangitits), liver fluke infection and anatomical abnormalities associated with biliary tract inflammation have been described, most occur in absence of known etiological factors (Ben-Menachem, 2007; Blechacz and Gores, 2008b).

The findings of CHC, particularly in patients of viral hepatitis B or C, indicate a common origin and/or shared pathogenic mechanisms for both HCC and ICC (Bhagat *et al*, 2006). Though several environmental carcinogenesis models of cholangiocarcinoma including infestation with liver flukes, especially the species *Opisthorchis viverrini* and *Clonorchis senensis*, have been established in animals, neither the etiological correlation nor any preclinical models associated viral hepatitis with ICC are available (Blechacz and Gores, 2008a).

Herein, we present our retrospective case–control studies on the association of viral hepatitis B and C with ICC. As at the time of HCC diagnosis, HCC patients with viral hepatitis B is ∼10 years younger than those with viral hepatitis C, we re-evaluated the association of viral hepatitis-related ICC with viral hepatitis-HCC. We compared the incidence and age distribution of ICC to that of HCC in patients with chronic viral hepatitis B or C, thereby contributing to our understanding of the pathogenesis of ICC and indirectly to that of HCC.

## Materials and methods

### Study subjects

The institutional ethics committee approved all protocols applied in this study. We selected consecutive cases of diagnosed ICC among inpatients who were admitted between 1991 and 2005 to our hospital, the Chang Gung Memorial Hospital at the Lin-Ko Medical Center. The criteria for case selection were the clinical and pathological diagnosis of ICC, with complete medical records for serum HBsAg (hepatitis B virus surface antigen) and anti-HCV (antibodies to hepatitis C virus), and pathological diagnosis, ultrasonography or other imaging studies to evaluate cirrhosis. Consequently, a total of 160 patients with ICC were included in this study.

Moreover, 2498 patients with HCC diagnosed at the same hospital during the same period from 1991 to 2005 were selected for the comparison.

Hospital controls matched for sex and age to the ICC patients were selected from individuals generally surveyed for any disease in our hospital.

To calculate the incidences of ICC, HCC and CHC in our country, we retrieved the case numbers of newly diagnosed ICC, HCC and CHC in Taiwan during 2003–2005 from our government cancer registration system (the Taiwan Cancer Registration System 2004–2006) (http://crs.cph.ntu.edu.tw).

### Liver cirrhosis

The criteria for the diagnosis of cirrhosis were as follows: clinical manifestations of chronic hepatitis with portal hypertension and/or hepatic decompensation, laboratory tests and hepatic ultrasonography. Characteristic signs included portal hypertension (varices, thrombocytopenia or splenomegaly) and/or signs of hepatic decompensation (jaundice, prolonged prothrombin time and ascites).

### Laboratory tests

Blood samples were tested for the surface antigens of hepatitis B virus (HBsAg) and antibodies against hepatitis C virus (anti-HCV) using enzyme-linked immunosorbent assays (Abbott Laboratories, North Chicago, IL, USA).

### Statistical methods

Independence tests were performed using unpaired *t*-test for continuous variables and χ-square or Fisher’s exact test for categorical variables. We calculated the OR (odds ratio), RR (relative risk) and PPV (positive predictive value) of ICC.

We used univariate logistic regression to determine the variables that predisposed to ICC, and then employed multivariate logistic regression without intercept to select the most important variable.

For each factor in the different populations, we also used logistic regression to estimate the OR and 95% CI (confidence interval) of the population-attributable risk, which was calculated using the OR of that factor and its prevalence in the control group.

## Results

### Patient characteristics

**Figure 1:**
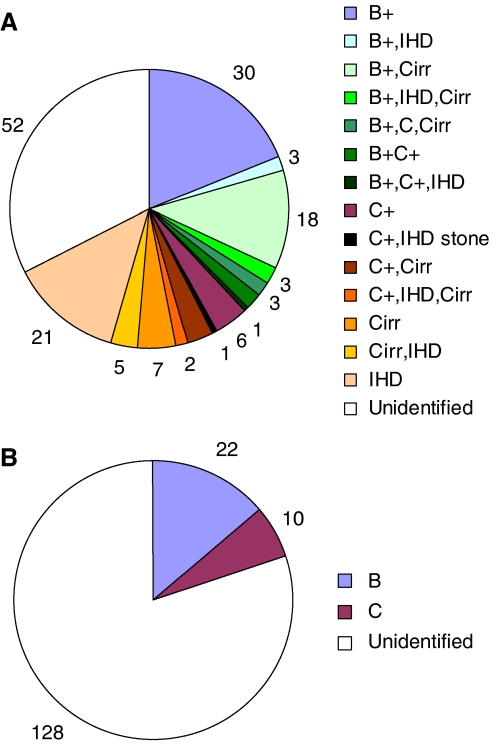
Distribution of potential risk factors for ICC among the 160 ICC patients (**A**) and the 160 control cases (**B**). B= seropositivity for HBsAg; C= seropositivity for anti-HCV antibody; Cirr= cirrhosis; IHD= intrahepatic duct stone.

We enrolled 160 consecutive patients of ICC and 160 age-matched individuals as controls to search the risk factors for ICC (Table 1 and Figure 1).

Of the 160 ICC patients, 60 were seropositive for HBsAg (37.5%); 21 were seropositive for anti-HCV (13.1%), including seven cases of seropositivity for both HBsAg and anti-HCV; 41 had intrahepatic-duct stones (IHD stones) (25.6%); and 21 had cirrhosis (25.6%) (Figure 1A). In the control group, 22 were seropositive for HBsAg (13.8%); 10 were seropositive for anti-HCV (6.3%) and none had IHD stone or cirrhosis (Figure 1B). In addition, patients with gallstone were seen in both the ICC group (18 patients, 11.3%) and the control group (22 patients, 13.8%) (Table 1), whereas patients with IHD stones (41 patients, 25.6%), cirrhosis (41 patients, 25.6%), and biliary parasite (*C. sinensis*) (1 patients) were only found in the ICC group (Table 1).

### Univariate analysis of the risk factors for ICC

As shown in Table 1, the sero-positive rates for HBsAg and anti-HCV in the ICC group were significantly higher than those in the control group (HBsAg: 37.5 *vs* 13.8%, *P*<0.001; and anti-HCV: 13.1 *vs* 6.3%, *P*=0.038). In addition, the prevalence of IHD stones and cirrhosis were also higher in ICC patients than in the controls (IHD stones: 25.6 *vs* 0%. *P*<0.001; and cirrhosis: 25.6 *vs* 0%, *P*<0.001). However, the difference in the prevalence of gallbladder stones or biliary parasite infection between the ICC and control groups was not statistically significant (gallbladder stones: 11.3 *vs* 13.8%, *P*=0.499; and biliary parasite infection: 0.6 *vs* 0%, *P*=0.317, respectively). It is worthy to mention that liver flukes, especially clonorchiasis and opisthorchiasis, which are prevalence in East and South Asia, have been proved to be the important causes of ICC. In our series, only one case of ICC had liver fluke. Low incidence of liver fluke infestation in our ICC patients could be explained by low prevalence of clonorchiasis in the past 13 years in Taiwan (<0.01% since 1995), though it was once very prevalent (about 1.5% in 1969; 0.2% in 1993 (Epidemiology Report, Division of Epidemiology, Department of Health, Taiwan, 1993; 9(5))).

To evaluate the contribution of each risk factor to ICC development, the ORs were analysed (Table 2). Patients with viral hepatitis B showed a 3.8-fold increase in the risk for ICC (95% CI: 2.17–6.53, *P*<0.001), and patients with viral hepatitis C showed a 2.3-fold increase in the risk for ICC (95% CI: 1.03–4.98, *P*=0.038). The PPV for ICC in the patients of viral hepatitis B and C were 18.13 × 10^−5^ and 10.92 × 10^−5^, respectively, which were also ∼three-fold higher than that in our general population (the prevalence of ICC in our population=2.48 × 10^−5^). As there were no cases of cirrhosis or IHD stones in the control group, neither the OR nor the PPV for ICC among patients of viral hepatitis B and C could be determined.

### Multiple logistic regression analysis

To determine the independent factor(s) in the development of ICC, multiple logistic regression analysis was conducted. As shown in Table 3, seropositivity for HBsAg, seroposivity for anti-HCV, and the presence of IHD stones were independent factors associated with ICC. Again, the OR’s for seropositivity for HBsAg and seropositivity for anti-HCV were 4.985 (95% CI: 2.775–8.945, *P*<0.001) and 2.709 (95% CI: 1.162–6.318, *P*=0.021), respectively.

### Synergistic effects of cirrhosis on the development of ICC in patients who were seropositive for HBsAg or anti-HCV

Cirrhosis is always associated with chronic inflammation either because of chronic B and/or chronic hepatitis C or because of bile duct inflammation, and ∼90% of HCC cases were detected in cirrhosis patients. Therefore, it is interesting to examine whether cirrhosis played a synergistic role in ICC development in patients with viral hepatitis B and/or C. Univariate logistic regression analysis of the effects of cirrhosis on these patients with chronic viral hepatitis showed that cirrhosis increased the risks for ICC by 2.5-, 3.2- and 12.6-fold in patients who were seropositive for HBsAg (95% CI: 1.196–5.095, *P*=0.015), anti-HCV (95% CI: 1.231–8.148, *P*=0.017) and both HBsAg and anti-HCV (95% CI: 2.533–62.923, *P*=0.002), respectively (Table 4).

### Age distribution of ICC *vs* HCC patients

**Figure 2:**
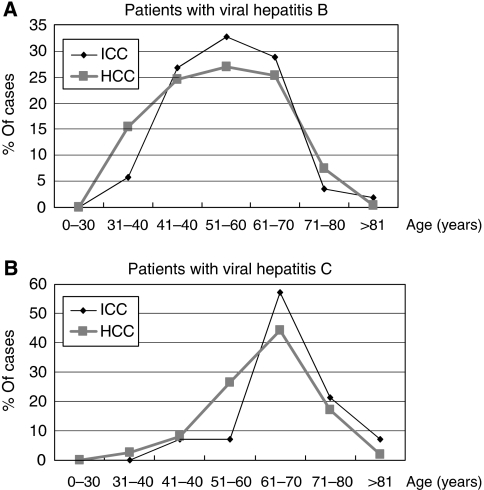
Comparison of disease incidence based on age distribution in the 160 ICC patients and 2498 HCC patients. (**A**) Profiles for viral hepatitis B patients with either HCC or ICC. (**B**) Profiles for viral hepatitis C patients with either HCC or ICC. Interestingly, the age distribution profiles of patients with viral hepatitis B and C are quite different in both ICC and HCC, whereas the age distribution profiles between ICC and HCC patients with either viral hepatitis B or C are nearly identical.

Strongly etiological association of chronic viral hepatitis and cirrhosis with ICC is reminiscent of that of HCC in our population. It is, therefore, tempting to speculate that viral hepatitis-associated ICC may develop through a similar pathogenic process to that for HCC development. Development of HCC in chronic viral hepatitis is a long-term process, and HCC patients of viral hepatitis B are usually about 10 years younger than HCC patients of viral hepatitis C, probably because of difference in the onset age of chronic viral hepatitis. If both viral associated HCC and ICC run through a similar pathogenic process, they would share similar difference of age distributions between patients with viral hepatitis B and C. To examine this hypothesis, we analysed age distributions of ICC in 60 patients with viral hepatitis B and 21 patients with viral hepatitis C (Table 5). Indeed, ICC patients with viral hepatitis B (56.4±11.1 years old) were about 9 years younger than ICC patients with viral hepatitis C (65.6±9.2 years old). We then compared the results to that of HCC in 2498 consecutive patients with HCC, who consulted our hospital during the period when ICC case collection (Figure 2). The mean age of HCC patients with viral hepatitis B (54.03±11.60 years) was about 9 years younger than that of HCC patients with viral hepatitis C (62.63±9.55 years). To further compare the details about onset age between ICC and HCC with the underlying viral hepatitis B *vs* C, the profiles of age distribution for both ICC and HCC were performed (Figure 2A and B). The profiles of age distribution between ICC and HCC patients with viral hepatitis B were basically consistent (Figure 2A). In addition, the profiles of age distribution between ICC and HCC patients with viral hepatitis C were consistent (Figure 2B). In contrast, the profiles of ICC and HCC patients with viral hepatitis B were greatly diverse from that of ICC and HCC patients with viral hepatitis C (Figure 2A vs B). In other words, the profiles of age distributions of viral hepatitis-associated ICC were consistent with those of viral hepatitis-associated HCC, suggesting the implication of common disease process for both viral hepatitis-associated ICC and HCC.

### The incidence of HCC, ICC and combined HCC with ICC in our population

Sharing common disease profiles for both viral hepatitis-associated ICC and HCC including viral-hepatitis etiology, synergic effects of cirrhosis and age distributions suggests the involvement of similar long-term disease process, and even a common cell origin for carcinogenesis. It is, therefore, intriguing to speculate that the relative incidences for HCC and ICC and the CHC might be related to the ratio of hepatocytes and cholangiocytes, both of which were differentiated from the hepatic progenitor cells (oval cells). We then calculated the relative incidences for HCC, ICC and CHC in our population. We retrieved the annular incidence of HCC, ICC and CHC from our national cancer registration system (the Taiwan Cancer Registration System, 2004–2006) (http://crs.cph.ntu.edu.tw). The new diagnosed cases of HCC, ICC and CHC in our population during the period 2004–2006 were 26 543 cases, 1949 cases and 114 cases, respectively. The case/number ratio of HCC : ICC : CHC was 233 : 17 : 1, which was consistent with the number ratio of hepatocytes to cholangiocytes in the liver.

## Discussion

Though association of viral hepatitis B and C with ICC has been documented (Wiwanitkit, 2005; Perumal *et al*, 2006; Shaib *et al*, 2007; Hsing *et al*, 2008; Zhou *et al*, 2008; El-Serag *et al*, 2009), the role of viral hepatitis B and C in the development of ICC in viral hepatitis endemic areas remains to be addressed. Herein, we not only show a strong etiological association of viral hepatitis B and C with ICC, but also provide circumstantial evidence to support the hypothesis of involvement of a common carcinogenesis process for both viral hepatitis associated-ICC and HCC. First, viral hepatitis B and C were independent risk factors and the major cause for ICC development. Our findings are consistent with that reported by El-Serag *et al* (2009)recently. Through a population based cohort study, El-Serag *et al* (2009) showed a strong association of HCC and ICC with HCV infection, but the association was not observed for extrahepatic cholangiocarcinoma. Second, as generally observed in HCC, cirrhosis – a pathological result of long-term chronic inflammation in the liver – exerted synergistic effects on ICC development in patients of viral hepatitis B and C. Third, both viral hepatitis-associated ICC and HCC had similar age profiles and difference in age distributions between patients with chronic hepatitis B and patients with chronic hepatitis C. Fourth, the ratio of disease incidences for HCC : ICC : CHC was consistent with the ratio of hepatocyte number to cholangiocyte number in the liver. These findings strongly indicated the involvement of similar long-term disease processes in the development of viral hepatitis-associated ICC and viral hepatitis-associated HCC.

It is known that chronic infection, for example, because of hepatitis B or C virus infection, plays a primary role not only in trans-activating cellular proto-oncogenes and/or disrupting tumour suppressor genes, but also in causing chronic inflammation. Sustained cell proliferation in a micro-environment rich in inflammatory cells, cytokines/chemokines, growth factors and DNA-damaging agents (such as reactive oxygen and nitrogen species because of long-term inflammation) will lead to permanent genetic alterations and subsequent neoplastic transformation of the proliferating cells (Komori *et al*, 2008). For example, interleukin-6, an inflammatory cytokine, promotes human cholangiocarcinoma cells grown *in vivo* by inhibiting apoptosis through the activation of miRNAs including miR let-7a and miR370, thereby modulating the activation of STAT-3 pathways (Meng *et al*, 2007, 2008; Smirnova *et al*, 2007). It has also been shown that the sustained upregulation of the transcription factor nuclear factor kappaB (NF-κB) in the liver cells, through the paracrine action of tumour necrosis factor-α secreted from the neighbouring endothelia and inflammatory cells, may lead to the tumour development (Maeda *et al*, 2005; Choi *et al*, 2006; Sakurai *et al*, 2006; Luedde *et al*, 2007), given the activation of mitogenic and anti-apoptotic genes through NF-κB pathways. In spite, the hypothesis of sharing common disease process for the carcinogenesis of viral associated ICC and HCC remains to be verified using cellular and animal models.

It has been known that men are about two–four times more likely to develop HCC than women (Bosch *et al*, 2004). Recent studies by using a chemical carcinogen (diethylnitrosamine) model of HCC in mice showed that the gender disparity in HCC was owing to suppression of interleukin-6 production in Kupffer cells by oestrogen (Naugler *et al*, 2007). Our studies showed a more prominence of gender disparity in HCC (male/female=2.91, the National Database, 2006) than in ICC (male/female=1.31, based on the National Database, 2006). In addition, the gender disparity in both HCC and ICC was even more prominent in patients with chronic hepatitis B (male/female=5.04 in HCC or 2.16 in ICC) than in patients with chronic hepatitis C (male/female=2.35 in HCC or 1.29 in ICC), suggesting that additional viral etiology and environmental factors also contribute to the gender disparity in both HCC and ICC development.

What are the target cells of chronic inflammation that induce similar carcinogenic processes for ICC and HCC in chronic viral hepatitis? Considering that tumour formation is a multi-step process involving the accumulation of mutations over years or even decades before the occurrence of neoplastic transformation, the target cells should be able to proliferate as well as survive in the liver for a long period. Some hepatocytes and bile duct cells (cholangiocytes) can temporally proliferate, and act as the target cells for carcinogenesis. The targeting of the proliferating cholangiocytes in the liver could explain why chronic inflammation of bile ducts, such as that caused by sclerosing cholangitis, hepatic fluke infection or hepatic lithiasis, is only associated with cholangiocarcinoma but not HCC. However, these proliferating cholangiocytes might not be the primary target cells for HCC development in patients with viral hepatitis B and C, unless trans-differentiation from the cholangiocyte to the hepatocyte lineage occurs. Similarly, proliferating hepatocytes can be the target cells for HCC. However, the requirement of a transitional differentiation process from the hepatocyte to the cholangiocyte lineage makes it less likely that viral hepatitis-associated ICC arises from the proliferating hepatocytes. More likely is that viral hepatitis-associated HCC and ICC are derived from the same cells of origin that have the potential to develop into both the hepatocyte and cholangiocyte lineages, and have long been exposed to the milieu related to genome injury and consequently, tumour transformation (Alison, 2006; Libbrecht, 2006; Nomoto *et al*, 2006; Roskams, 2006; Zhang *et al*, 2008). Indeed, it is now generally believed that cancer can originate from stem cells or progenitor cells rather than from differentiated cells as these are the only cells that persist in the tissue for a sufficient length of time to acquire the requisite number of genetic changes for neoplastic transformation (Alison and Lovell, 2005).

The speculation that both HBV- and HCV-associated HCC and ICC are derived from liver progenitor cells (oval cells) is further supported by the occurrence of CHC, in which both the histological components of HCC and ICC are found simultaneously. Recently, it has been suggested that CHCs may arise from hepatic progenitor cells that retain their potential to differentiate into the hepatocytic and biliary lineages (Libbrecht, 2006; Zhang *et al*, 2008).

Hepatic progenitor cells (oval cells), residing in the canal of Hering in the liver, are multipotent progenitor cells that can give rise to both hepatocytes and cholangiocytes. They represent the replicating cellular compartment after liver injury. The degree of reactivation of hepatic progenitor cells has been associated with the degree of inflammatory activity in viral hepatitis and cirrhosis (Roskams *et al*, 2003; Roskams, 2006). Recently, cells with stemness properties have been identified in human HCC, further supporting the speculation that hepatic progenitor cells are involved in the carcinogenesis of HCC as well as ICC (Barajas *et al*, 2007; Ma *et al*, 2007; Yamashita *et al*, 2007; Zen *et al*, 2007; Qiao *et al*, 2008; Yang *et al*, 2008; Zhang *et al*, 2008).

The reason why the incidence of ICC in patients of viral hepatitis B or C is substantially lower than that of HCC if both arise from the same hepatic progenitor cells with a common pathogenic process remains unknown. Our explanation is that the incidence of HCC *vs* ICC is determined by the ratio of the number of the progenitor cells differentiating into the two lineages hepatocytes *vs* cholangiocytes. Indeed, hepatocytes represent approximately 80–90% of the liver cells, whereas bile duct cells account approximately 5–7% of the liver cells. If this is the case, the incidence of HCC : ICC : CHC should be approximately 225 : 15 : 1 (p : q : pq, where p and q are the incidences for HCC and ICC, or the relative numbers of hepatocytes and cholangiocytes in the liver, respectively). Consistent with this estimation, the incidence of HCC : ICC : CHC in our population was 233 : 17 : 1 (HCC : ICC : CHC=26543 cases : 1949 cases : 114 cases=233 : 17 : 1) (according to the Taiwan Cancer Registration System, 2003–2005) (http://crs.cph.ntu.edu.tw). Further studies should be undertaken to determine the roles and mechanisms of hepatic progenitor cells in neoplastic transformation into HCC as well as ICC in patients of viral hepatitis B or C.

We conclude that besides other causes of bile duct inflammation, viral hepatitis B and C are the primary independent risk factors for ICC in our country, a viral hepatitis endemic area. Cirrhosis exerts synergistic effects on ICC development in patients of viral hepatitis B or C. Patients with viral hepatitis B developed ICC ∼9 years earlier than patients with viral hepatitis C did. The incidence of ICC by age distribution in patients with either viral hepatitis B or C was nearly identical to that of HCC in patients with viral hepatitis B or C. The ratio of the incidence of HCC to ICC to CHC might reflect the ratio of the numbers of hepatocytes and cholangiocytes differentiated from the same hepatic progenitor cells. Our findings suggested that both viral hepatitis-associated ICC and HCC originate from the same hepatic progenitor cells through a similar long-term inflammatory carcinogenic process. Our findings also suggest that all risk factors causing chronic inflammation of the liver including chronic viral hepatitis, inflammatory bile duct disease, hepatic lithiasis and hepatic parasite infection could activate a common pathway for the development of liver malignancy. Moreover, suppression of inflammation may reduce cellular dysplasia, thereby preventing malignancy.

## Figures and Tables

**Table 1:** Demographics and baseline clinical characteristics of ICC patients and control cases

	**ICC (*n*=160)**	**Control (*n*=160)**	***P*-value**
Gender (male/female)	101/59	87/73	0.112
Age (mean±s.d., years)	61.5±12.0	61.0±12.2	0.765
HBsAg	60 (37.5%)	22 (13.8%)	0.000
Anti-HCV	21 (13.1%)	10 (6.3%)	0.038
			
*Biliary stones*			
GB stones only	18 (11.3%)	22 (13.8%)	0.499
IHD stones	41 (25.6%)	0	0.000
			
Liver cirrhosis	41 (25.6%)	0	0.000
Biliary parasites	1	0	0.317

HBsAg=seropositivity for hepatitis B surface antigen; Anti HCV=seropositivity for anti-hepatitis C virus antibody; GB=gall bladder; IHD=intrahepatic ducts.

*P*-values were determined by univariate logistic regression, *χ*^2^- test.

**Table 2:** Univariate analysis of risk factors at presentation that were associated with the ICC (160 patients) and control (160 persons) groups

	**HBsAg**	**Anti-HCV**	**Cirrhosis**	**IHD stones**
*Positive rate*				
ICC patients	37.5%	13.1%	25.6%	25.6%
Controls	13.8%	6.3%	0%	0%
Odds ratio	3.763	2.266	—a	—a
*P*-value	0.000	0.038	0.000	0.000
PPV	18.13 × 10^−5^	10.92 × 10^−5^		
95% CI	2.17–6.53	1.03–4.98	—a	—a

PPV=positive predictive value; HBsAg=seropositivity for hepatitis B surface antigen; anti-HCV=seropositivity for anti-hepatitis C virus antibody; IHD=intrahepatic duct.

*P*-values were determined using *χ*^2^ or Fisher’s exact test as any of the value <5.

The annular incidence of cholangiocarcinoma was 4.819 × 10^−5^ in our general population. PPV=OD × annular incidence.

a No case of IHD stones or liver cirrhosis was observed in the control group. Therefore, the odds ratio and 95% CI (confidence interval) could not be calculated.

**Table 3:** Multiple logistic regression analysis of risk factors for ICC

	**Odds ratio**	**95% CI**	***P*-value**
HBsAg (1)^a^	4.985	2.775–8.945	0.000
Anti-HCV (1)^a^	2.709	1.162–6.318	0.021
IHD stone	4.810	2.626–8.015	0.000
Cirrhosis	—b	—b	—b

HBsAg=seropositivity for hepatitis B surface antigen; anti-HCV=seropositivity for anti-hepatitis C virus antibody; IHD=intrahepatic duct.

a Variable(s) entered in step 1: HBsAg, anti-HCVand IHD stones.

b No case of liver cirrhosis was found in the control group; therefore, the odds ratio and 95% CI (confidence interval) could not be calculated.

**Table 4:** Logistic regression in 74 cases of viral associated ICC and the synergistic effects of liver cirrhosis on viral hepatitis B and C

**Risk factors**	**Additional factors**	**No.**	***P*-value**	**Odds ratio**	**95% CI**
*HBsAg*					
With	Cirrhosis	38	0.015^*^	2.468	1.196–5.095
Without		22			
					
*Anti-HCV*					
With	Cirrhosis	11	0.017^*^	3.167	1.231–8.148
Without		10			
					
*HBsAg+Anti-HCV*					
With	Cirrhosis	3	0.002^*^	12.625	2.533–62.923
Without		4			

HBsAg=seropositivity for hepatitis B surface antigen; Anti-HCV=seropositivity for anti-hepatitis C virus antibody; 95% CI=95% confidence interval. ^*^P<0.05 was considered significant (two-sample t-test).

**Table 5:** Comparison of age at diagnosis amongst different groups of ICC patients

	**ICC**		
**Factors**	**Case no.**	**Age: Mean±s.d.**	***P-*value**
*HBsAg*			
+	60	56.4±11.1	0.000^*^
−	100	64.6±11.3	
*Anti-HCV*			
+	21	65.6±9.17	0.096
−	139	60.9±12.3	
*IHD stone*			
+	59	64.9±11.8	0.005^*^
−	101	59.5±11.7	
*Cirrhosis*			
+	41	59.8±10.8	0.306
−	119	62.1±12.3	
			
All cases	160	61.5±12.0	

Anti-HCV=anti-hepatitis C virus antibody; HBsAg=hepatitis B surface antigen.

+/–: seropositivity/seronegativity.

^*^P<0.05 was considered significant (two-sample *t*-test).
